# The Role of Trap-assisted Recombination in Luminescent Properties of Organometal Halide CH_3_NH_3_PbBr_3_ Perovskite Films and Quantum Dots

**DOI:** 10.1038/srep27286

**Published:** 2016-06-01

**Authors:** Zhen-Yu Zhang, Hai-Yu Wang, Yan-Xia Zhang, Ya-Wei Hao, Chun Sun, Yu Zhang, Bing-Rong Gao, Qi-Dai Chen, Hong-Bo Sun

**Affiliations:** 1 State Key Laboratory on Integrated Optoelectronics, College of Electronic Science and Engineering, Jilin University, 2699 Qianjin Street, Changchun, 130012, People’s Republic of China; 2 Center of Interface Dynamics for Sustainability, Institute of Materials, China Academy of Engineering Physics, 596 Yinhe Road, Chengdu 610200, People’s Republic of China

## Abstract

Hybrid metal halide perovskites have been paid enormous attentions in photophysics research, whose excellent performances were attributed to their intriguing charge carriers proprieties. However, it still remains far from satisfaction in the comprehensive understanding of perovskite charge-transport properities, especially about trap-assisted recombination process. In this Letter, through time-resolved transient absorption (TA) and photoluminescence (PL) measurements, we provided a relative comprehensive investigation on the charge carriers recombination dynamics of CH_3_NH_3_PbBr_3_ (MAPbBr_3_) perovskite films and quantum dots (QDs), especially about trap-assisted recombination. It was found that the integral recombination mode of MAPbBr_3_ films was highly sensitive to the density distribution of generated charge carriers and trap states. Additional, Trap effects would be gradually weakened with elevated carrier densities. Furthermore, the trap-assisted recombination can be removed from MAPbBr_3_ QDs through its own surface passivation mechanism and this specialty may render the QDs as a new material in illuminating research. This work provides deeper physical insights into the dynamics processes of MAPbBr_3_ materials and paves a way toward more light-harvesting applications in future.

The past few years have witnessed the erupted research of solution-processed organic-inorganic metal halide perovskite. These low-cost semiconductors materials have exhibited not only tunable absorption and emission across the visible spectrum^1^,^2^,^3^,^4^,^5^,^6^,^7^,^8^,^9^,^10^, but also intriguing charge-transport properties especially the ultra-long charge-carrier diffusion length^11^,^12^,^13^,^14^,^15^,^16^,^17^,^18^. Perovskite devices with high power-conversion efficiency in architectures have been proposed such as mesoporous films^19^,^20^,^21^, planar films^22^,^23^,^24^,^25^, single crystals^9^,^26^,^27^,^28^,^29^, and colloidal quantum dots (QDs)^1^,^2^,^5^,^30^. Due to the relative smaller exciton binding energy in perovskite materials, it has been confirmed that the band-edge recombination was dominated through charge carriers (non-geminate electrons and holes), accompanied by minor Auger recombination (charge carriers mode) which occurred at very high pump fluences^11^,^13^. More over, in the published results^11^,^14^, it is found that the lifetimes of these charge carriers in transient absorption (TA) spectroscopy measurements are fluence-dependent. In crystallography, defects exist at the lattice grain boundaries and gaps of individual crystals in a crystal film^31^,^32^,^33^ leading to the presence of the trap-assisted recombination mode. More recently, enormous correlative proofs about trap states have been reported. The Snaith group confirmed that Bright electroluminescence (EL) and photoluminescence (PL) can only be achieved under enough high excited intensity or time^34^. We previously reported that the intensity and lifetime of charge carriers decreased in impurities doped CH_3_NH_3_PbI_3-x_Cl_x_ perovskite films^35^. The Ginger group illustrated that the trap states quenched crystalline fluorescence of discontinuous perovskite films by measuring the microscopic PL^33^. The Ma group demonstrated that the kinetic traces in the vicinity of lattice grain boundaries was very different by microscopic TA spectrum measurements^36^.

The trap-assisted recombination mode will decrease the integral density and lifetime of the charge carriers, deteriorating the optical performances of perovskite films. To date, chemical passive treatments with external materials (such as pyridine, thiophene and iodopenta-fluorobenzene) have been succeeded proposed to remove the trap-assisted recombination process^2^,^33^,^37^. Despite the certain amount of passive methods, there are still relatively scarce reports on the fundamental photophysics of the trap-states^11^,^12^,^13^,^14^,^15^,^16^,^17^,^18^. The results show that at low pump fluences, the integral TA and PL kinetics traces were combined by trap-assisted and charge-carrier recombination modes. The trap states of films caused great loss in intergral fluorescence intensity and lifetime. The trap effect could be gradually eliminated with the elevated density charge carriers, additionally, when the density of charge carriers far exceed that of trap states, the films kinetics was dominated by charge carrier recombination mode. At last, we proposed an effective strategy of using MAPbBr_3_ QDs to remove the trap effect due to its own surface passivation mechanism^1^. Its surface capping ligands can effectively repair the trap states, leading to the integral decay kinetics can be dominated by charge carrier recombination mode.

## Results and Discussion

**Figure 1:**
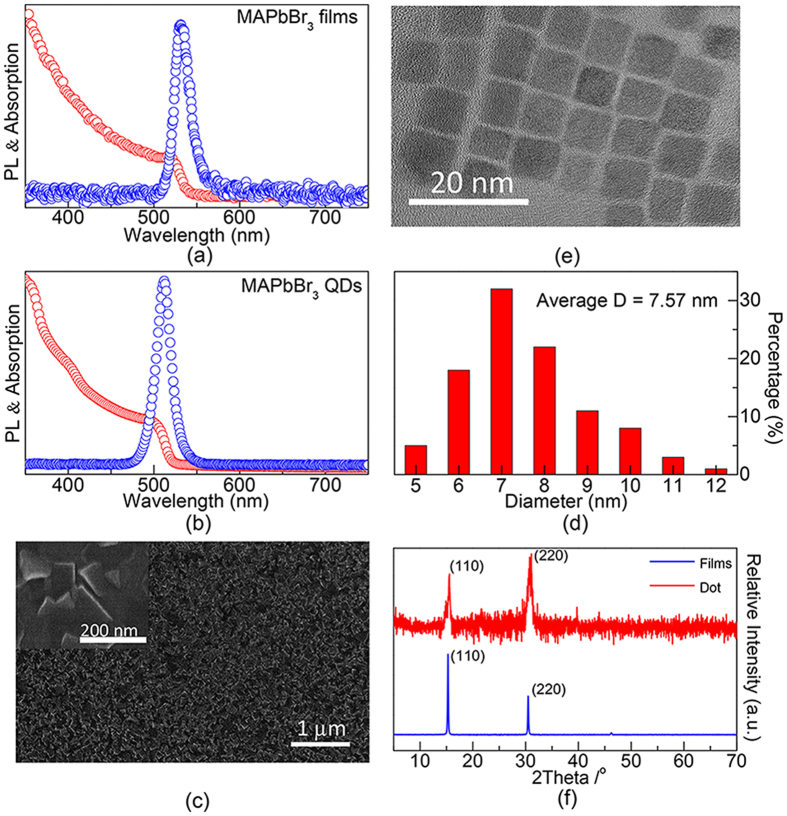
UV-vis absorption and PL emission spectrum of (**a**) MAPbBr_3_ films and (**b**) MAPbBr_3_ QDs. (**c**) Top-view SEM images of integral MAPbBr_3_ films and several grains respectively, the scale bar was 1 μm and 200 nm. (**d**,**e**) Show the TEM image as well as the sizes histograms of MAPbBr_3_ QDs respectively. The scale bar was 20 nm and the average diameter was 7.57 nm. (**f**) XRD patterns of MAPbBr_3_ films and QDs.

The measured UV-vis absorption (red) and the PL spectrum (blue) of MAPbBr_3_ films as well as QDs are illustrated in Fig. 1a,b respectively. In Fig. 1a, absorption of MAPbBr_3_ films has a band edge at ~520 nm. A sharp emission peak at ~530 nm with fwhm (full width at half maximum) value of only 23 nm (~96 meV) can also be readily observed. Relatively smaller Stokes shift of ~49 meV implies that the PL emission of MAPbBr_3_ perovskite films originates from direct-gap recombination. The absorption shoulder and PL peak of MAPbBr_3_ QDs can be read as 505 and 515 nm respectively in Fig. 1b. Compared with Fig. 1a,b, PL emission of MAPbBr_3_ QDs was found ~20 nm (95 meV) blue-shifted compared to that of MAPbBr_3_ bulk materials. The observed spectra blue shift could be explained by the proposed quantum confinement effect in a reported work^1^. Morphological investigation by scanning and transmission electron microscopy (SEM and TEM) were performed for MAPbBr_3_ films and QDs respectively. The top-view SEM image in Fig. 1c shown that the MAPbBr_3_ crystalline grains nearly full covered the substrate and the sizes of the grains reads ranging from 100–150 nm. The scale bar was 1 μm. Figure 1d,e respectively had shown a typical TEM image of MAPbBr_3_ QDs as well as their sizes distribution. The scale bar was 20 nm. The TEM image confirmed the MAPbBr_3_ QDs crystallized well and the columnar distribution map shown that the QDs have an average diameter of 7.57 nm with size deviation of ±0.7 nm. Aimed to analyze the phase structure of the MAPbBr_3_ system, as illustrated in Fig. 1f, X-ray diffraction (XRD) spectrum was performed. The peaks at 15.34^o^ and 30.48^o^ are correlated to the (110) and (220) diffractions of MAPbBr_3_ crystals respectively.

**Figure 2:**
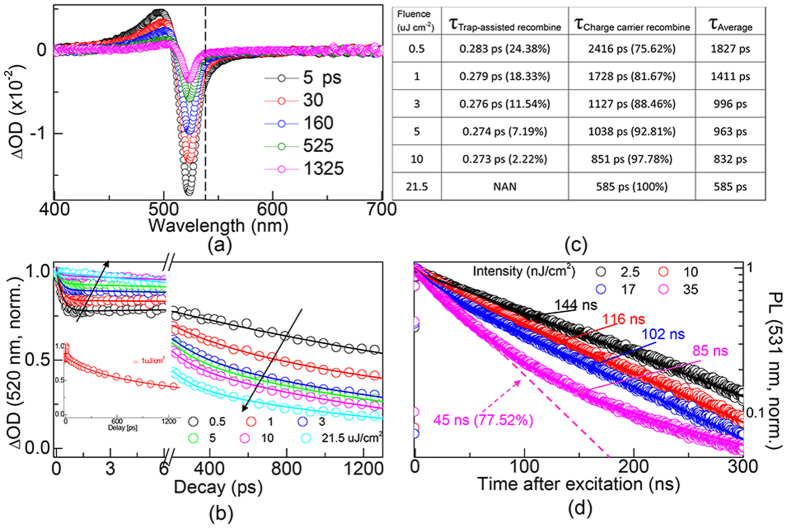
(**a**) Time-resolved difference absorption spectra of MAPbBr_3_ films which were pumped under 400 nm with intensity at 7.5 μJ cm^−2^. Laser induced fluorescence was marked by dash line. (**b**) Fluence-dependent normalized kinetic traces of band-edge (520 nm) transition. The arrows indicate direction of increasing pump intensities ranging from 0.5 to 21.5 μJ cm^−2^. (**c**) Lifetimes and corresponding ratios of trap-assisted and charge carrier recombination mode, the average lifetime shows decreased trend with increased pump fluences. (**d**) Fluence-dependent time-resolved PL measurements of MAPbBr_3_ films with 405 nm pulses, trap-assisted kinetics trace was marked by dash line.

To understand the role of trap effect in the charge carriers’ properties of perovskite films, investigation on the transient recombination processes were performed using a fs-TA setup. Figure 2a shows the time-resolved difference absorption spectra of MAPbBr_3_ films which were pumped under 400 nm with intensity at 7.5 μJ cm^−2^. From the TA spectra, there are three main features: a photoinduced transient bleach at ~520 nm, a photoinduced excited state absorption (EAS) in the range of 450–500 nm and an additional transient signal at ~530 nm. Compared with the steady-state absorption and PL spectrum, the signals at 520 and 530 nm (marked by dash line) can be, respectively, assigned to transient bleaching of the band edge transition and laser-induced fluorescence. Figure 2b shows the fluence-dependent normalized kinetic traces of band-edge (520 nm) transition. The pump intensities range from 0.5 to 21.5 μJ cm^−2^ and arrows indicate increasing directions. Consistent with the proposed theory, charge carrier recombine process was inevitably influenced by trap-states due to trap-filling effect^34^. As illustrated in Fig. 2b, integral kinetic traces are split, showing former part was trap-assisted recombination mode and the later part was charge carrier recombination mode. The inset shows the dynamics under 1 μJ cm^−2^ without delay break. It was clear that the rates of charge carrier recombination accelerate with elevated pump fluences. TA dynamics (lines) were modeled as a combination of trap-assisted recombination and charge carrier recombination modes. In Fig. 2c, we report on the fitted lifetimes and ratios of the two part combinations under different pump fluences. The lifetimes of trap-assisted recombination are almost fluences independent, raning from 0.283 to 0.273 ps, and the ratios decreased from 24.38% (pump fluence: 0.5 uJ cm^−2^) to 0% (21.5 uJ cm^−2^) with increased pump fluences. On the other hand, the lifetimes of charge carrier recombination through electrons and holes are highly sensitive to the pump fluences, decreased from 2416 (pump fluence: 0.5 uJ cm^−2^) to 585 ps (21.5 uJ cm^−2^), accompanied by increased ratios from 75.62–100%. The integral average lifetime decreased from 1827 to 585 ps. It can be learned that the trap effect can be gradually weakened when more electrons and holes are generated under elevated pump intensities, leading to the charge carrier recombination mode become dominating. The PL decay trace was confirmed by a single-channel time-correlated single-photon counting (TCSPC) system with pump at 405 nm and the fluence-dependent PL kinetics of MAPbBr_3_ films was shown in Fig. 2d. It was clear that the rates of integral recombination increased with elevated pump fluences ranging from 2.5 to 35 nJ cm^−2^. PL dynamics were also modeled (lines) as a combination of trap-assisted recombination and charge-carrier recombination modes. From Fig. 2d, at low pump fluences (black, red and blue lines), PL kinetics are dominated by trap-assisted recombination exhibiting nearly single exponential decay with fluence-dependent lifetimes of 144 ns (pump fluence: 2.5 nJ cm^−2^), 116 ns (10 nJ cm^−2^) and 102 ns (17 nJ cm^−2^). At a relative higher fluence (35 nJ cm^−2^), traps are predominantly full filled by photoinduced generated partial charge-carriers. As remarked above, the PL recombination (magenta line) under high pump fluence was composed by trap-assisted recombination and charge-carriers recombination modes, exhibiting double exponential decay with integral lifetime of 85 ns. Two different time scales were assigned to the trap-assisted part (fast) together with charge-carrier part (slow), fitted by a fast lifetime of 45 ns at ratio 77.52% and a slow lifetime of 231 ns at ratio 22.48%. Trap-assisted recombination kinetics traces were modeled by dash lines.

**Figure 3:**
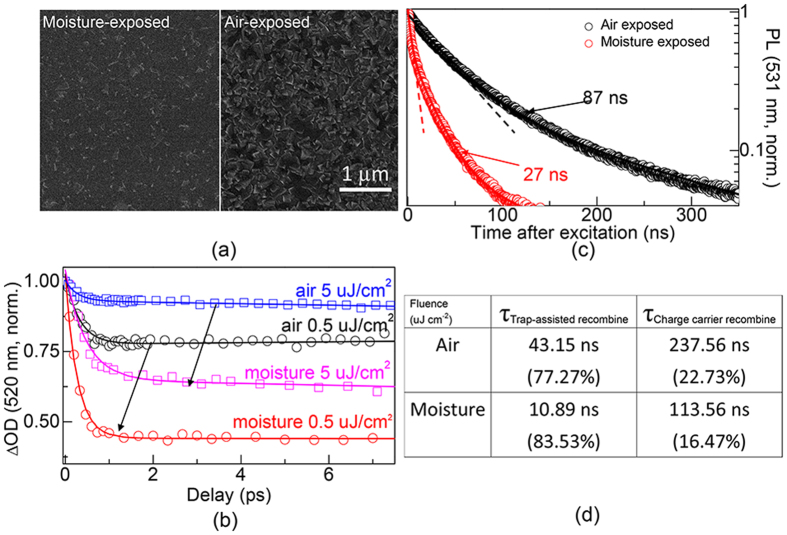
(**a**) SEM images of the MAPbBr_3_ perovskite layer exposed to moisture (left) and air (right). (**b**) Normalized TA kinetic traces of air- and moisture-exposed MAPbBr_3_ films under identical pump fluences. The arrows indicate direction of increasing trap-states densities. (**c**) Time-resolved PL measurements of these two samples, trap-assisted kinetics traces were marked by dash lines. (**d**) Lifetimes and corresponding ratios of trap-assisted as well as charge carrier recombination mode for air- and moisture-exposed MAPbBr_3_ films.

The role of exposed environment on the morphology of the MAPbBr_3_ perovskite films were examined by SEM images (Fig. 3a). The scale bar was 1 μm. There was a drastic change on the morphology of MAPbBr_3_ crystalline grains due to degradation effect^35^ when films are exposed to moisture (50%, maintain 5 s). It can be easily observed that the fraction of coverage in air-exposed MAPbBr_3_ films (right) was much higher than that of moisture-exposed films (left), moreover, the shape of the individual crystallites was also much better defined. Moisture-exposed MAPbBr_3_ crystallites cannot form continuous dense networks but rather scattered grains with lager gaps, indicating the presence of much more trap states. The absorption (red line) and PL spectrum (blue line) of moisture-exposed films are shown in Fig. S1 of the Supporting Information. Figure 3b compares the normalized TA kinetic traces of air- and moisture-exposed MAPbBr_3_ crystallites under identical pump fluences. Due to the increased gaps in moinsture-exposed films, the fitted ratios of trap-assisted recombination mode increased from 24.38–56.92% (circle, pump fluence: 0.5 uJ cm^−2^) and 7.19–35.25% (square, 5 uJ cm^−2^). The arrows indicate direction of increasing trap densities. It can be speculated that the larger gaps in moisture-exposed MAPbBr_3_ films could result in more loss of integral fluorescence lifetime and intensity. Figure S2 of the Supporting Information shows the decreased fluorescence intensities in these samples. In moisture-exposed perovskite film, more surface are exposed to environments when the concentrated grains are broken by moisture, the enhanced surface induced the generation of smaller band gaps according to solide state physics, resulting into the fluorescence red-shift. Shown in Fig. 3c, the PL dynamics (lines) were modeled as a combination of trap-assisted recombination and charge-carriers recombination modes exhibiting compound exponential with integral lifetime decreased from 87 to 27 ns (fluence: 35 nJ cm^−2^). Trap-assisted recombination kinetics traces were modeled by dash lines. Furthermore, In Fig. 3d, we report the lifetimes and ratios of the two components in these two films. When the exposed environment converted from air to moisture, the ratios of trap-assisted recombination increased from 77.27–83.53% accompanied by the lifetime decrease from 43.15 to 10.89 ns. Meanwhile, the ratios of charge carrier recombination decreased from 22.73–16.47% and the corresponding lifetime decreased from 43.15 to 10.89 ns. It can be learned that, the PL performance of perovskite was high sensitive to the density of trap states, and the result indicates that these materials should be prior isolated from massive moisture.

**Figure 4:**
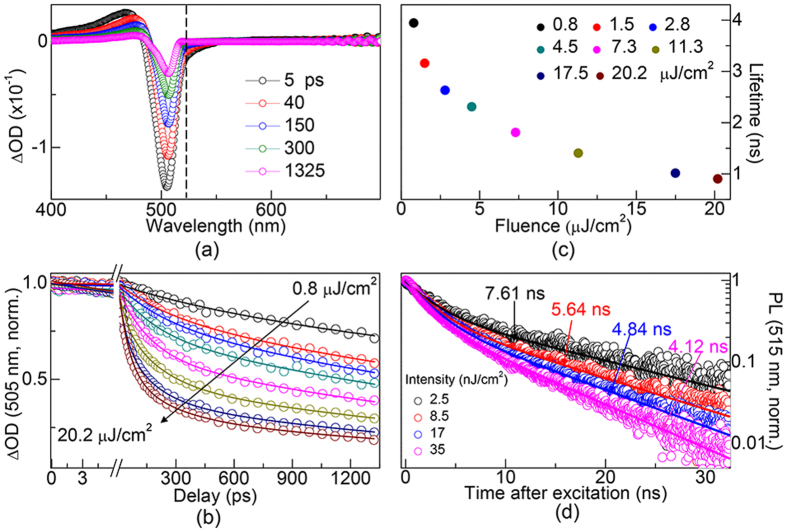
Time-resolved difference absorption spectra of MAPbBr_3_ QDs which were pumped under 400 nm with intensity at 7.5 μJ cm^−2^. The dash line indicates laser induced fluorescence. (**b**) Fluence-dependent normalized kinetic traces of band-edge (505 nm) transition. The arrows indicate direction of increasing pump intensities ranging from 0.8 to 20.2 μJ cm^−2^. (**c**) Lifetimes show decreased trend with elevated pump fluences at decreasing rates. (**d**) Fluences-dependent time-resolved PL measurements of MAPbBr_3_ QDs with 405 nm pulses.

Although MAPbBr_3_ perovskite exhibit better advantage than many other materials^38^,^39^,^40^, they still suffer from great loss of PL intensity and lifetime induced by trap-states. It was likely that one could optimize MAPbBr_3_ optical performance by removing the trap-assisted recombine process. Here, we report a passivation strategy of using MAPbBr_3_ QDs to overcome the problem of trap effects, this was because the QDs can passivate the trap-states through the proper surface chemical capping ligands *n*-octylamine during its synthesis process. The surface capping ligands *n*-octylamine covered the surface of QDs and can effectively passive surface trap states. Figure 4a shows the time-resolved difference absorption spectra of MAPbBr_3_ QDs which were pumped under 400 nm with intensity at 9.4 μJ cm^−2^. Three main features can be readily observed: a photoinduced transient bleach at ~505 nm, a photoinduced ESA in the range of 400–480 nm, and an additional transient signal at ~515 nm. Similar, the signals at 505 and 515 nm (marked by dash line) can be assigned to band edge transient bleaching and laser-induced fluorescence, respectively. Figure 4b shows the fluence-dependent normalized kinetic traces of band-edge (505 nm) transition. Arrows indicate the pump intensities increased from 0.8 to 20.2 μJ cm^−2^. It was clear that the trap-assisted kinetics traces was non-existent in QDs compared with that of films shown in Fig. 2b. The TA dynamics were modeled (lines) as charge-carriers recombination mode and the rates increased with elevated pump fluences. Figure 4c shows the fitted fluence-dependent carriers’ lifetimes of band-edge transition as a function of pump fluences and the lifetime decreased from 3944 to 902 ps at decreasing rates^11^. This results was the indicative of well passivation effect in QDs, leading to the elimination of trap effect. The fluence-dependent PL decays of MAPbBr_3 _QDs are shown in Fig. 4d. The two PL decay time and ratios of the decays are listed in S3 of Supporting Information. It was clear that recombination rates increased with elevated pump fluences ranging from 2.5 to 35 nJ cm^−2^. PL dynamics (lines) were modeled as charge-carriers recombination, exhibiting exponential decay with lifetimes decreased from 7.61 ns (fluence: 2.5 nJ cm^−2^) to 4.12 ns (35 nJ cm^−2^) with elevated pump fluences.

## Conclusion

In the present work, we provided a comprehensive study of time-resolved relaxation dynamics in MAPbBr_3_ films and QDs utilizing time-resolved PL as well as TA spectroscopy. The results have shown that the trap-assisted recombination mode existed in MAPbBr_3_ films resulted in negative effects for the charge carrier performance of perovskite. The TA and PL results show that the trap states changed integral photocarrier recombination mode and lead to an obvious decrease in the integral fluorescence lifetime and intensity. Trap effects can be removed by a strategy of using colloidal MAPbBr_3_ QDs for the chemical passivation of QDs surface capping ligands can effectively weaken trap effect. The fitted fluence-dependent TA and PL revealed that the QDs dynamics was dominated by charge carrier recombination mode. These results provide deeper insights into the underlying photophysics in MAPbBr_3_ materials and such acknowledge are necessary for the optimization of highly efficient emitting devices in future.

## Methods

MAPbBr_3_ films synthesis: Firstly, 0.421 mmol MABr and 0.141 mmol PbBr_2_ were simultaneously dissolved in dimethylformamide DMF (2 ml) forming precursor solution (wt ~5%). Then the precursor solution (200 μl) was spin coated on the glass using spin coater at 3000 rpm. At last, the precursor films were settled on the thermal platform (~80 ^o^C), after thermal annealing for 20 min, the precursor films has been crystallized to MAPbBr_3_ perovskite lattice films. All the experimental procedures were performed in a nitrogen filled glove box.

MAPbBr_3_ perovskite QDs synthesis: Firstly, 0.33 mmol MABr, 0.38 mmol PbBr_2_, 1 ml oleic acid and 0.04 ml n-octylamine were loaded into DMF (8 ml) forming precursor solution. Then 2 ml precursor solution was siphoned and then injected in to toluene solution (10 ml), At last, the QDs formed after violent stirring for 2 min. Aggregated colloidal QDs (yellow-green) were separated by centrifuging at 10000 rpm for 5 min, and then dissolved in toluene forming stable solutions protected by N_2_.

Steady spectrum characterization: Perovskite extinction coefficients and PL spectrum were examined by a Shimadzu UV-2550 spectrophotometer a Hitachi F-4600fluorescence spectrophotometer, respectively. XRD patterns were got from a Rigaku X-ray diffractometer.

TA spectrum system: In fs-TA system, a Ti: sapphire laser (Newport Corporation, USA) outputs 100 fs pulses of 795 nm wavelength at 250 Hz repetition rate. The laser beam was split, 25% laser energy was focused into sapphire sheet forming probe beam ranging from 460 to 800 nm. The probe light was recorded by a highly sensitive spectrometer (Avantes AvaSpec-2048 × 14). The other laser energy was penetrated through an optical chopper forming pump beam at 125 Hz repetition. The TA signals were recorded by controlling relative time delay between pump and probe beam by optical delay line.

PL spectrum system: This was a single-channel time-correlated single-photon counting (TCSPC) system. A picosecond diode laser (Edinburgh Instruments EPL405) outputs 405 nm wavelengths at 2 MHz repetition rate. Samples were excited at 405 nm, the fluorescence signals were selected by a monochromator and amplified by a photomultiplier tube (Hamamatsu H5783p). The kinetics was recorded at TCSPC board (Becker and Hickel SPC-130).

## Additional Information

**How to cite this article**: Zhang, Z.-Y. *et al*. The Role of Trap-assisted Recombination in Luminescent Properties of Organometal Halide CH_3_NH_3_PbBr_3_ Perovskite Films and Quantum Dots. *Sci. Rep.* **6**, 27286; doi: 10.1038/srep27286 (2016).

## Supplementary Material

Supplementary Information
