# Supplementary material for: The Role of Trap-assisted Recombination in Luminescent Properties of Organometal Halide CH3NH3PbBr3 Perovskite Films and Quantum Dots
Source: Sci Rep. 2016 Jun 1;6:27286. doi: 10.1038/srep27286 (PMC4888750; doi:10.1038/srep27286)
Supplement: Supplementary Information [file srep27286-s1.pdf]

Supporting Information

The Role of Trap-assisted Recombination in Luminescent Properties of Organometal Halide $CH_3NH_3PbBr_3$ Perovskite Films and Quantum Dots

Zhen-Yu Zhang[1], Hai-Yu Wang[1], Yan-Xia Zhang[1], Ya-Wei Hao[2], Chun Sun[1], Yu Zhang[1], Bing-Rong Gao[1], Qi-Dai Chen[1], and Hong-Bo Sun[1]

[1]State Key Laboratory on Integrated Optoelectronics, College of Electronic Science and Engineering, Jilin University, 2699 Qianjin Street, Changchun, 130012, People's Republic of China.

[2] Center of Interface Dynamics for Sustainability, Institute of Materials, China Academy of Engineering Physics, 596 Yinhe Road, Chengdu 610200, People's Republic of China.

Correspondence and requests for materials should be addressed to H.Y.W. (haiyu_wang@jlu.edu.cn) or H.B.S (hbsun@jlu.edu.cn)

Zhen-Yu Zhang	1105680976@qq.com
Hai-Yu Wang	haiyu_wang@jlu.edu.cn
Yan-Xia Zhang	1511235040@qq.com
Ya-Wei Hao	394898628@qq.com
Chun Sun	842214702@qq.com
Yu Zhang	yuzhang@jlu.edu.cn
Bing-Rong Gao	492377231@qq.com
Qi-Dai Chen	11989974@qq.com
Hong-Bo Sun	hbsun@jlu.edu.cn

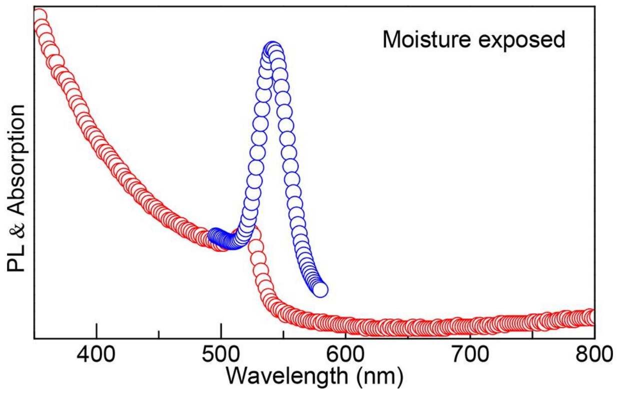

Figure S1. UV-vis absorption and PL emission spectrum of moisture-exposed $MAPbBr_3$ films. The relative larger defects density cause the band-edge transition converted from shoulder to peak.

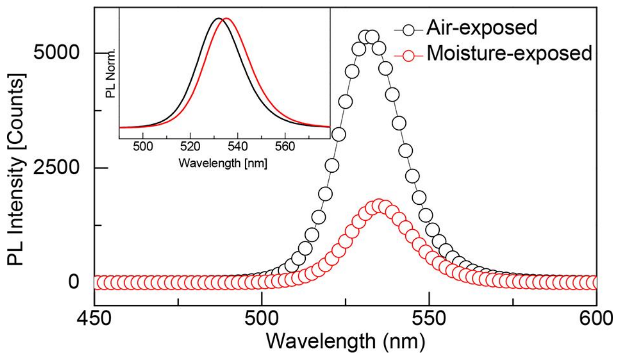

Figure S2. Contrastive PL emission spectra of air- and moisture-exposed $MAPbBr_3$ films. The fluorescence intensity of degraded $MAPbBr_3$ films decreased and with slight red shift (inset).

Fluence (uJ cm^{-2})	τ_1 (ns)	τ_2(ns)	$\tau_{average}$(ns)
2.5	2.45(57.48%)	14.59(42.52%)	7.61
8.5	2.38(64.16%)	11.48(35.84%)	5.64
17	2.21(65.51%)	9.84(34.49%)	4.84
35	2.05(66.22%)	8.18(33.78%)	4.12

Figure S3. The fluence-dependent PL decay times and ratios of perovskite QDs.
